# Renovating the Barnes maze for mouse models of Dementia with STARR FIELD: A 4-day protocol that probes learning rate, retention and cognitive flexibility

**DOI:** 10.1101/2024.11.30.625516

**Published:** 2024-12-01

**Authors:** Aimee Bertolli, Oday Halhouli, Yiming Liu-Martínez, Brianna Blaine, Ramasamy Thangavel, Qiang Zhang, Eric Emmons, Nandakumar S. Narayanan, Serena B Gumusoglu, Joel C. Geerling, Georgina M Aldridge

**Affiliations:** a Department of Neurology, Carver College of Medicine, University of Iowa; b University of Iowa Department of Obstetrics and Gynecology, University of Iowa; c Iowa Neuroscience Institute

**Keywords:** long term memory, short term memory, working memory, perseverance, perseveration, neurodegeneration, executive function, normal pressure hydrocephalus, traumatic brain injury, aging, behavioral flexibility, maze, radial arm

## Abstract

Land-based mazes that require spatial cues to identify the location of a hiding-place are a low-stress method to evaluate learning rate and memory retention in mice. One version, the Barnes maze, allows quantification of naturalistic exploratory behaviors not evident in water-based tasks. As the task relies on innate behaviors, it does not require overtraining, making it more feasible to examine early learning and non-memory executive functions that are characteristic of some non-amnestic dementias. However, because it is difficult to hide odor cues in the traditional version of the maze, learning rate during individual trials can be difficult to interpret. We designed and tested the use of 3D-printed escape shuttles that can be made in duplicate, as well as a docking tunnel that allows mice to self-exit the maze to improve reproducibility and limit experimenter influence. In combination with maze turning and escape tunnel caps, we show our shuttles mitigate the possibility of undesired cues. We then compare use of our 4-day protocol across several mouse models of cognitive impairment. We demonstrate an additional stage, the STARR protocol (Spatial Training and Rapid Reversal), to better challenge executive functions such as working memory and behavioral flexibility. We examine commonly used outcome measures across mice with and without access to spatial cues, as well as across mouse models of cognitive impairment to demonstrate the use of our 4-day protocol. Overall, this protocol provides detailed instructions to build and perform a robust spatial maze that can help expand the range of deficits identified. Our findings will aid in interpretation of traditional protocols, as well as provide an updated method to screen for both amnestic and non-amnestic cognitive changes.

## INTRODUCTION

Neurodegeneration and dementia affect 55 million people worldwide [1]. Mouse models are used for studying mechanisms and testing new therapies; they have similar cortical architecture, many conserved brainstem projections compared with humans, and abundant available genetic tools [2; 3; 4; 5]. Studies in rodents have spurred progress in understanding mechanisms of short-term memory deficits in Alzheimer’s dementia (AD). However, fewer studies focus on non-memory aspects of cognition, such as executive dysfunction, that are common features in other forms of dementia, including frontal variants of AD, Lewy Body Dementias, Frontotemporal dementias, vascular dementia, traumatic brain injury and normal pressure hydrocephalus [6; 7; 8; 9].

Behavioral assays that use innate drives like novelty exploration and memory of an escape location (novel object recognition, Morris Water Maze) do not require extensive training and are relatively easy to interpret, allowing rapid evaluation [10; 11; 12]. However, many use short-term memory (or evidence suggestive of memory) as the key outcome measure, even when researchers are trying to study non-amnestic disorders or learning rate [13; 14]. Non-memory behaviors, such as executive functions (e.g., working memory, behavioral flexibility, sequencing, planning) and motivations (e.g., perseveration, perseverance, exploration, anxiety) can be key features in both amnestic and non-amnestic neurodegenerative diseases [15; 16].

Behavior variability makes studying executive functions difficult in mice. Mice behavior is very sensitive to manipulation, which can be beneficial for evaluating treatments, but leads to increased group variability. Manipulations can impact motivation or motor movements, induce use of alternative strategies and cues or alter anxiety in ways that impact outcomes [17; 18; 19; 20; 21; 22]. Studies probing executive functions often require large animal numbers, long training protocols, overtraining, expensive operant chambers or strong motivators, each creating potential caveats [18].

For these reasons, we sought to evaluate and modify a commonly used test of amnestic function and spatial learning to screen for a wider range of executive, learning, psychiatric and motivational outcomes that encompass both amnestic and non-amnestic disorders. We chose to optimize the “Barnes Maze” a spatial learning and memory task originally developed by Carol Barnes for rats [23]. This task has significant beneficial features that allow it to be used with a variety of dementia and neurodegenerative models and is used in hundreds of published research articles yearly. Specific benefits of the Barnes Maze are that it: 1) resembles innate naturalistic behavior in rodents (exploration and remembering hiding locations [18; 24]), 2) does not require food or water motivation, 3) is comparatively low stress, 4) can be adapted for mice with motor deficits because it avoids forced swimming, 5) and is commonly available and relatively inexpensive to build [25; 26].

During the Barnes maze, mice are placed in the center of a large open field with holes around the outer edge, and with one of the holes leading to a dark escape box. Mice have an innate desire to find escape routes and remember their location [12]. Tests using “looming” of a predator-like cue show that after just a single trial, mice remember the spatial location of a hide-out when in the presence of a predator-like cue [27; 28]. In the Barnes maze, since highly aversive cues like a predator or water-swimming are not used, multiple trials are used to reinforce the location of the hide-away. Typically, changes in distance, latency, or number of explored holes needed during learning trials to find the escape are used as a measure of learning speed, while the percentage of time near the hole during a probe trial represents short or long-term spatial memory.

During optimization of this task, we noted that olfactory cues represent a major theoretical confound during typical use, potentially impacting learning trials and could inadvertently hamper spatial memory should the mouse focus on the wrong cue. Most laboratories and commercial devices use a single escape box during all learning trials, which then becomes a potential source of olfactory cues. Many studies also do not describe turning the maze or moving the visual cues, instead relying on washing the maze. While investigating potential motivators early in our evaluation, we found that if the mouse’s home-cage was attached to the escape box (out of sight), mice could repeatedly find the target hole without approaching any other hole, even when the target was moved to a random spatial location every trial. In typical laboratory use, the escape box is usually cleaned each trial and thus represents a much more subtle potential cue. However, this theoretical concern remains, which is why many researchers favor the probe trial (when the escape box is absent) for interpretation.

An issue with reporting only the probe trial is we lose essential information about learning rate, search strategy, and motivation, decreasing our sensitivity to detect differences between manipulations or groups. We also cannot evaluate trial-by-trial changes occurring following reversal learning to test behavioral flexibility. We therefore designed an inexpensive “3D-printable” escape box that could be deployed on most standard Barnes mazes. This allows any researcher to have multiple copies of the escape box to eliminate olfactory cues as a potential source of information for the mouse. We have taken this strategy to its theoretical limit: we created a version of the Barnes maze that can be made for less than the cost of the commercial task, but with the benefit of having an identical escape box at every hole. Entry to incorrect holes is blocked with plastic grates made from the same type of plastic, and each individual escape box or hole is not used more than once per 10 trials.

Here, we demonstrate testing and use of these 3-D printed spatial learning “shuttle-boxes” that allow analysis of learning trials without the theoretical confound of potential olfactory cues. We name this adapted protocol the FIELD protocol (**F**ind the **I**nvisible **E**xit to **L**ocate the **D**omicile), aka Barnes-FIELD, due to two key features: 1) **Exits** are “invisible” due to the use of caps (to hide visual cues) and shuttle-copies (to hide olfactory cues). 2) Shuttles are designed to allow a direct link to the home-cage (Domicile), connected after the exit is located, providing a naturalistic reinforcement cue with reduced experimenter interference.

Our study comparing the FIELD protocol to unwashed shuttles demonstrated mice were able to detect an olfactory cue based on probe trial outcomes, but olfactory cues did not improve latency on learning trials compared with random chance. The design of the study allowed us to demonstrate and confirm prior literature [29; 30; 31] showing that the most noticeable gains in latency and distance during early learning trials can be explained by non-spatial learning (procedural, motivational, etc.) rather than spatial learning.

By adapting the Barnes to incorporate intermittent probe trials within the learning trials, we demonstrate the timing of acquisition of spatial memory during early and late training, and screen for motivational differences.

Finally, we present an additional stage, the **STARR** FIELD protocol (**S**patial **T**raining and **R**apid **R**eversal), which can be incorporated at the end of any common Barnes protocol (including our Barnes FIELD protocol) to screen for behavioral flexibility. We evaluate the use of the Barnes FIELD and STARR FIELD protocols in several different amnestic and non-amnestic models of dementia and aging, highlighting aspects of the task that can be evaluated when all trials can be confidently interpreted using our method.

## MATERIALS AND METHODS:

### Equipment:

#### Shuttles and docking:

**Figure 1:**
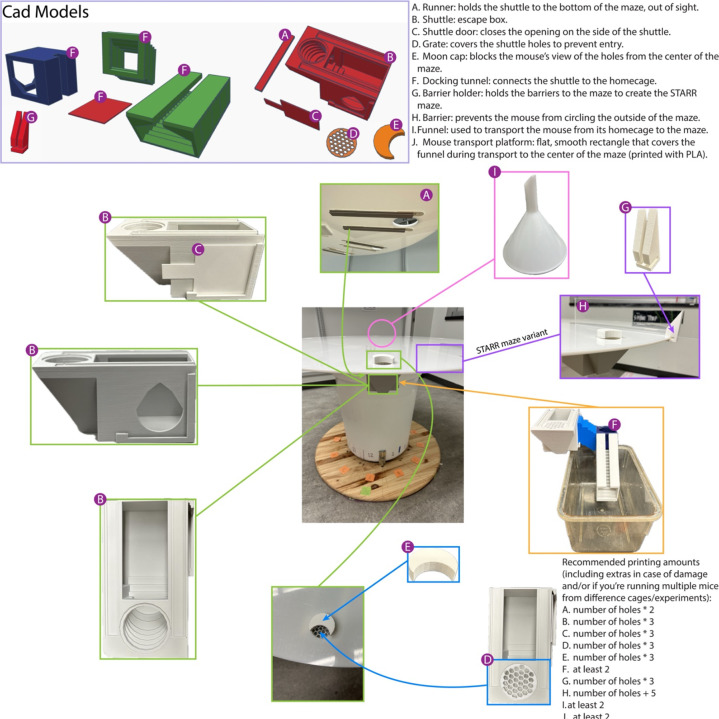
STARR and FIELD equipment: (A) 3-D printable runners, (B) 3-D printed shuttles, (C) 3-D printed doors, (D) 3-D printed blocking grates, (E) 3-D printed moon caps, (F) 3-D printed docking tunnel, (G) 3-D printed barrier holder, (H) Plastic, transparent barrier (measurements in STARR Maze section), (I) Release Funnel, (J) 3-D printed mouse transport platform (any thin rigid sheet can be used).

Shuttles, docks and most associated materials can be 3D printed. For this study, all items indicated as 3-D printed were printed on a Prusa i3 MK3S+ without supports (Fig 1). All devices were printing using Polylactic acid (PLA). Grey PLA (AmazonBasics, Color: Light Grey, 1.75mm) was used for shuttles based on early testing: white let in too much light to the shuttle boxes and darker colors interfered with tracking. White PLA (AmazonBasics, Color: White, 1.75mm) was used for caps, doors, grates and barrier holders. STL files to print these items are available at TinkerCad (Autodesk) opensource for further adaptation. Links to all printable items are available at aldridge.lab.uiowa.edu/open-science. The type of PLA used for printing is no longer available, thus any PLA or PLA+ of light grey and white, respectably, should be usable. Identical items should be printed with the same material.

The shuttle, cap, grate, and runners: Can be printed without supports. No assembly is required.

The docking tunnel (Fig 1F): This is printed in three pieces and glued with Loctite^®^ Instant Mix Epoxy (47oz).

Runners are assembled under the open platform using command strips (Command^™^ Large Picture Hanging Strips) allowing removal of the runners if necessary.

Recommended numbers of printed pieces are listed in Figure 1. To assemble, place the runners into the shuttles then add a command strip on each end of each runner. Allow to set per manufacturer guidelines. Then remove backing from other side of command strip and place on the underside of the maze aligning the opening of the shuttle with the 2-inch hole. Press firmly. The shuttle should then slide easily onto and off the maze like a drawer for cleaning and replacement.

#### Platform and rotating maze:

A commercial Barnes maze cost thousands of dollars and usually includes only a single escape box. However, many labs already own this apparatus or parts of one that can be adapted with our methods. The original maze available to our laboratory (brand unknown) had only the white plastic platform: 122 cm (~48 inches) in diameter, 0.635 cm (1/4 inch) thick, with 40 holes (2 inches (5.08 cm in diameter)), 5.397 cm (2.125 inches) from the edge, equally spaced around the perimeter. It was missing all other parts. For the proposed version of the task all but 10 holes were taped shut from the underside. For ease of cleaning and budget, we recommend investigators that do not have their own maze to custom order the top out of HDPE plastic, with the preferred number of holes from any plastic manufacturer (Supplemental Fig 1). Based on our internal testing of 8 vs. 10 vs. 12 vs. 20-hole mazes, and published data in the literature [20] we did not find improved discrimination ability with increased hole numbers. Past a certain point (e.g., 32 holes) mice tend to find the maze much more difficult requiring more learning trials and/or switching to serial search strategies [32]. When building from scratch, we therefore recommend making a maze with 12 holes located and named by the hands of a clock (Supplemental Figure 2), as this allows for statistical convenience to divide into quadrants for probe trials, with one target hole and two adjacent holes in a sector.

The plastic platform is then fixed firmly on top of a clean, white, plastic, 55-gallon container (33” tall, 26.5” wide (FG265500WHT, Grainger)). Although not required, a smoothly rotating maze with fixed locations will provide a much more reproducible maze set up. At the minimum, locations should be taped/marked on the floor and the stand to allow reproducible turning of the maze (Fig. 1). In our setup, the 55-gallon container was secured to a RAM-PRO 12-Inch Heavy Duty Rotating Swivel Turntable/lazy Susan (Amazon). The turntable was then fixed onto a 48” diameter wood tabletop (Menards). A sliding lock was bolted onto the white container such that it could be locked into any of 10 fixed, drilled holes. Care should be taken to align and fix (e.g., glue) the turntable, container, and maze-platform in a position such that the maze turns symmetrically, and none of the maze is cutoff in the video. Otherwise, software-based analysis will be more difficult as the hole will shift slightly each time the maze is rotated. While some laboratories move only the visual cues instead of rotating the maze, our preference is to allow multimodal spatial learning (sound, vibration, external odors, light gradients, etc.) of the external spatial environment for a more naturalistic task and quicker acquisition.

### Room setup:

The testing area should be quiet, well lit, containing discrete visual cues in all directions. Smaller items in the room should be marked to keep them in reproducible locations across time, including any cleaning equipment. A monitoring area in close proximity to the testing area but behind a curtain (Proman Products Room Divider, Black [SKU: B07VJXTK87]) is useful to prevent interference by movements of the experimenter, with the experimenter always remaining in the same location behind the curtain. Flood lights (4 in total) were purchased from Amazon (Neewer Dimmable Bi-Color LED [Amazon]). Note that some flood lights flicker depending on the camera’s frame rate.

70% ethanol can be used between trials on the maze to reduce odor cues. Although the maze is rotated, odor cues from prior trials can theoretically confuse the mice and so effort should be made to wipe the maze during the interval. All shuttles and other items should be cleaned with appropriate cleaning agent per local animal care guidelines between mice. PLA plastic did not deform when cleaned with Alconox, 70% ethanol, or a hydrogen peroxide based cleaner (Rescue). We used 1% Alconox, Rescue and water rinse for washing shuttles, caps, grates, and tunnels, followed by air drying.

The maze is set up by covering all decoy shuttles with grates, placing shuttles under every open hole, placing doors on the shuttles, sliding the shuttles underneath all active (target and decoy) holes, and covering the holes with caps facing toward the center of the maze (Figure 1). Set up all additional items in labeled locations to ensure reproducibility. This includes the release funnel (should be opaque/frosted), extra clean shuttles and shuttle attachments (doors and grates), clean moon caps, escape tunnel, the home cage, which will hold one subject at a time, and an extra clean and empty cage if the mouse has cage-mates. Finally, you will need a cart to sit the home cage on during docking, sized appropriately so the tunnel can reach and allow the mouse entrance into the cage, and a drape to cover the home-cage with after attaching the docking tunnel to the shuttle at the end of a trial.

### STARR-FIELD protocol add-on setup:

For the STARR-FIELD (4^th^ day) protocol, the room and maze are identical to that described above except 10 plastic barriers are added to create a clear radial arm maze the encourages the mouse to return to center after each hole is checked. Barriers are inexpensive and can be custom ordered from any plastic supplier: 0.250 thick clear cast acrylic paper-masked sheet, Size: 16“ × 3.5” (Professional Plastics, Sacr.250CCP). Barrier holders are 3-D printed and will fit on a maze 0.635 cm thick.

### Mice:

All experimental procedures were performed in accordance with relevant guidelines by the University of Iowa Institutional Animal Care and Use Committee (IACUC). Mice were housed 12:12 light:dark, and run on the maze during day hours. For experiment one testing the shuttles, male and female C67BL/6J (#000664) were purchased from the Jackson Laboratory at 3 months of age and started at least 1-week following arrival.

### The 4-day Barnes-FIELD protocol:

#### Overview:

The protocol consists of four days of training and testing, with 4 separate probes to evaluate memory at different points in learning (early and late) and lengths of retention (short and long). The first day, Day 1, starts with a habituation trial during which the bedding from the home-cage is placed in the escape shuttle to encourage standardized acclimation without the interference of the examiner that is common during typical mouse protocols. This is followed by four spatial acquisition trials on Day 1.

Day 2 starts with a probe trial (early-training, 24h memory) that is followed by six spatial acquisition trials.

Day 3 starts with another probe trial (late-training, 24h memory), followed by six spatial acquisition trials, and a final, short-term memory probe (late training, ~3min memory). The 3-min probe is a positive control (should be the easiest).

**Figure 2:**
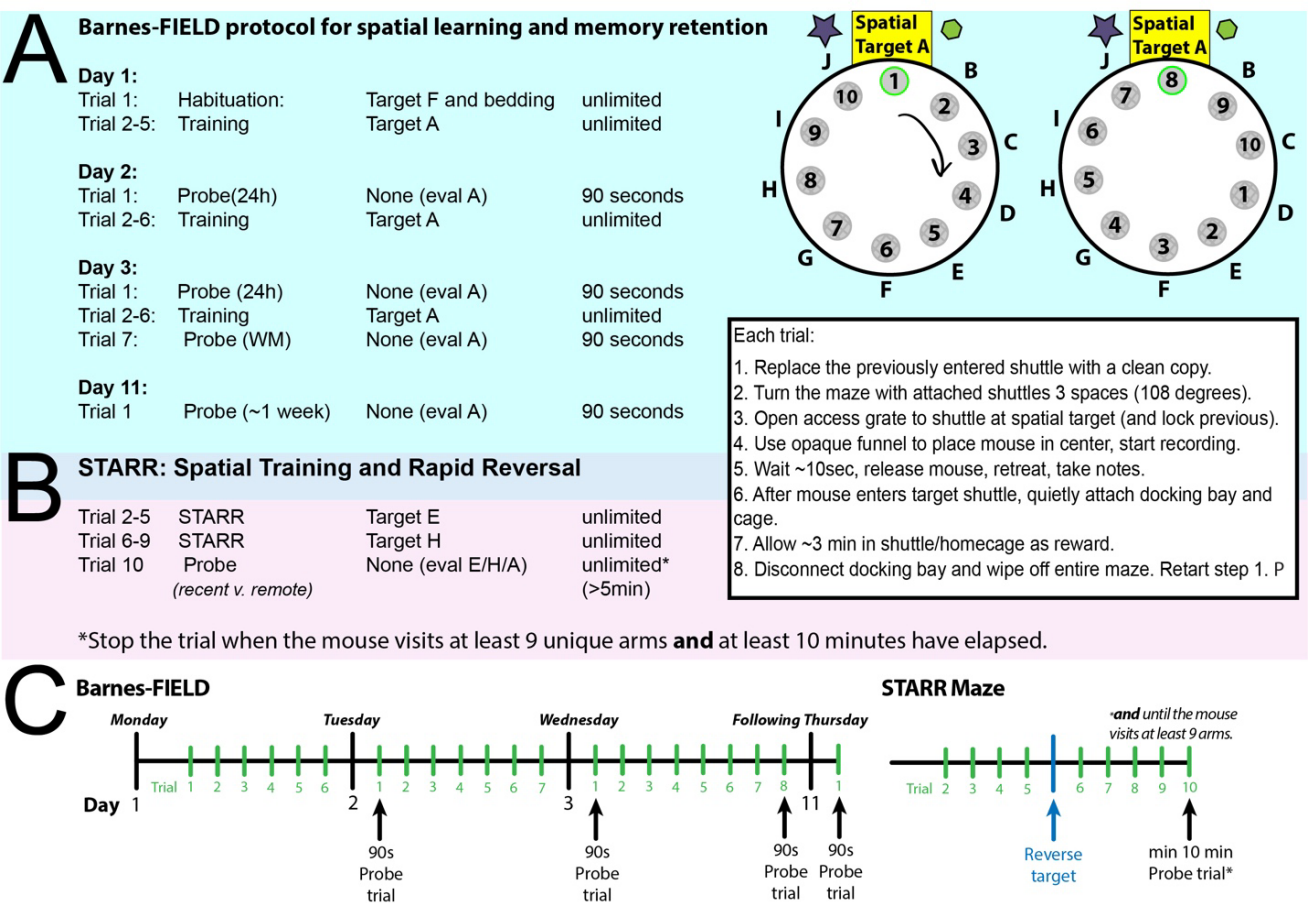
Printable Short Form Protocol (A) The 4-day Barnes-FIELD Protocol for spatial acquisition and short and long-term memory in a 10-hole version. (B) The STARR Protocol to test behavioral flexibility. (C) Overall schematic of both with example days.

Approximately 1 week following training completion (Day 11, counted as the fourth day of the protocol) mice run a single probe trial (late training, ~1week memory probe). We used day 11, such that the maze could be run Monday-Tuesday-Wednesday and the following Thursday each week (Fig 2). This allows new cohorts of mice to be run every week without overlap. The 1-week probe can be followed immediately by the optional STARR-FIELD protocol (below) to test exploration efficiency, working memory and behavioral flexibility.

All trials have an inter-trial interval of three minutes. We recommend all learning trials have unlimited duration to improve statistical power, but some severely impaired mice may require an adaptation (see trouble shooting). Probe trials are conducted with all escape shuttles blocked and have limited duration of 90 seconds except as noted. A step-by-step protocol is described below:

### Barnes FIELD Procedure: Step-by-step

1. Don lab coat and gloves. Make all needed cleaning solutions. Turn on flood lights. Preclean maze. Fully set up all shuttles with one shuttle below each hole, and all holes blocked by grates except the target hole. A moon shaped cap should block each hole from visual view of the center. Turn on video recording and test maze rotation alignment. Set up naming scheme for files. Write name of mouse on white board in field of view of camera.
2. The mouse should start in its original home cage. Cage-mates should be placed in a separate clean cage away from the task until testing is complete.
3. Using the starting funnel and mouse transport platform, place the mouse in the center of the maze, covered by the funnel. Gently slide out the platform taking care not the pinch the mouse’s tail.
4. Start the video recording and wait 10 seconds. Then, lift the funnel and step out of the testing area promptly. Monitor the mouse by video and wait until the mouse enters the target hole. Remain quietly in the same position at all times during the trial.
5. *Only for probe trial:* wait the listed time, adding 10 seconds if using the video recording clock and then remove the mouse. Use the funnel/platform in reverse to remove the mouse. Use caution during the probe trials not to handle the mouse roughly as this may increase variability, causing increased fear-related learning in some mice.
6. Stop the video recording.
7. For training trials: Once the mouse enters the target, cover the target hole with a cap to prevent the mouse from leaving its shuttle. Bring the home cage into the testing area. Attach the shuttle to the home-cage via the docking tunnel and cover the home-cage with a drape to further incentivize the mouse’s return. Remove the shuttle door to allow the mouse entrance to the home-cage.
8. While the mouse is getting “rewarded” (either sitting in the shuttle or making its way down to the home cage), wipe the maze with 70% ethanol solution. Once you’re done cleaning the maze and the mouse is in the home cage, disconnect the docking-tunnel from the shuttle and home cage, and remove the shuttle from the maze. Roll the cart/home-cage out of the training area and back to its idle position. Clean the shuttle per protocol and put it to dry. Choose an unused, clean, dry shuttle to put back under the maze.
9. Rotate the maze 108° (3 holes) clockwise. Remove the grate from the shuttle that is now at Spatial Location A (for this example*). Make sure all other shuttles are blocked and fully pushed into position. This takes about 2–3 minutes (inter-trial interval). Using the starting funnel, place the mouse in the center of the maze, covered by the funnel. Repeat steps 3–9 above for each trial.
10. For subsequent mice: Wash the maze with an appropriate agent. Counterbalance your targets across experimental groups (2 is sufficient, greater numbers will lead to more human error). Write the target on the white board for each mouse run.

### Trouble shooting:

1. If the mouse falls or jumps off the apparatus during testing, **keep recording**; promptly enter the testing area and return the mouse to the center of the apparatus.
2. If the mouse is resistant to leaving the shuttle, remove the tunnel and place the entire shuttle (mouse still inside), inside the home cage. Usually, the mouse will then leave on their own or can be gently coaxed out.
3. If the mouse is resistant to leave the docking-tunnel. Place the entire tunnel in the cage. If they do not then leave on their own, the tunnel is designed with openings to allow the mouse to be coaxed out.
4. Shuttle drying: Printing ~30 shuttles allows the shuttles time to dry between mice, allowing continuous running. It is recommended that you print additional copies of all items (~2–3 escape tunnels), >30 copies of all other items (caps, grates, shuttle doors).
5. Mice cannot find the shuttle: Typical protocols recommend stopping the trial after 3 minutes [24] if the mouse fails to find the target. Unfortunately, ending the study due to a set cutoff time prevents use of “distance” or “errors” as a primary outcome because a mouse that doesn’t move much will have a “short” (better) distance. For these reasons we recommend allowing mice unlimited time to locate the target. However, we have found some mice fall asleep on the maze when they fail to find the target. In these cases, we have used experimenter encouragement to allow the mice to complete the maze. This is not ideal (experimenter involvement increases variability and potential bias), but we found on balance provides slow or anxious mice the opportunity to learn the spatial target. We recommend the following protocol if intervention is needed or time must be limited:
  - Wait 10 min before intervening if mouse is actively exploring.
  - If after 5 min the mouse stops moving (presumed sleep), for >2 min, walk into the room. If the mouse returns to exploring, leave the room.
  - If the mouse does not move, use the mouse transfer platform to encourage the mouse back to the center of the maze. It is important statistically that the investigator does NOT encourage the mouse to the target hole.
  - Repeat step b-c each time the mouse falls asleep.
  - Continue until the mouse has visited all 10 holes. If the mouse doesn’t enter once they have visited the target, it is then permissible to encourage the mouse into the target hole. (The trial ends when the mouse has visited the target, even if they have not entered).

Of note: Failure to find the target hole is a rare occurrence in most mice but is expected for some severe phenotypes or occasionally when running a large number of mice. As with all studies, the experimenter must use some judgement in adapting the protocol to severe phenotypes to avoid misattributing motivational, exploration or mood abnormalities to cognitive (memory, learning, attention, etc.) deficits.

### Additional Notes on the recommended protocol:

#### Moon caps:

In early testing of our protocol with a preliminary cohort of mice with a strong behavior phenotype (a 5xFAD cross), we found the mice could learn to push a cap off the hole when it was completely blocked, but would often “forget” how to push the cap by the following day, halting any further trials (data not shown). This deficit prevented the further pursuit of using fully covered holes to improve statistical analysis of our task; Acquisition of “pushing caps” required 2 days of training and the experimental mice could not retain this training. Instead, this led to our use of the current moon shaped caps to block visual cues, but does not require pushing to enter the escape hole. Although we have removed the most difficult aspect (pushing the caps), the task still requires non-spatial learning (search around the caps) that may be the primary deficit for some models.

#### Sound cue:

We have avoided a loud sound, used in some version of the Barnes, due to risk of increased experimenter error (timing of turning off cue when hole entered) as well as experimenter fatigue and annoyance.

### Spatial Training and Rapid Reversal (STARR) Protocol: Overview

Immediately following the standard 1-week probe trial, barriers can be added to the open platform to create a clear radial-arm style maze (Fig 1, Fig 8J). If desired, the STARR-FIELD protocol can be run alone in one day if mice complete at least one habituation trial first without the barriers; however, skipping the initial Barnes misses information about spatial acquisition and long-term memory/retention, and there is an increased chance of mice jumping the barriers or hiding next to them if they are not fully trained using the standard protocol.

### STARR Procedure: Step-by-step

1. Run either the 1-week probe or a habituation trial for naïve mice as trial 1 (no barriers).
2. Add the barriers, using the barrier holders.
3. Follow steps 1–9 of the Barnes FIELD procedure with the following notable changes:
4. For trials 2–5, the spatial target is set at E for this example*.
5. The maze is turned 108 degrees (clockwise 3 holes in trials 2–4). After trial 5, do not turn the maze (the target location will change instead).
6. For trials 6–9, the spatial target is set at H, for this example*. After each of trials 6–9, turn the maze 108 degrees (counterclockwise, 3 holes).
7. For trial 10: Run a probe trial with the barriers all in place. IMPORTANT: Stop the trial when the mouse visits **at least 9 unique arms** and **at least 10 minutes have elapsed**. This gives both an exploration distance over a set interval, and a statistical estimate of number of working memory errors over a set amount of explored maze.

*Notice from Figure 2 that each target for the STARR has two adjacent holes/arms beside them that have not been used for target acquisition. You do not have to use the configuration of AEH (and counter-balancing is recommended), but choose targets that will not overlap.

#### Data Analysis:

Data can be analyzed from videos manually (by hand) or with computer software. Outcome measures are each discussed below, including when they can be extracted manually for those with limited budget. The investigator analyzing the data should be blind to treatment condition regardless of method. In this study we used AnyMaze (Ver. 6.36 and 7.48), EthoVision (Ver. 14 and 17) and cross-validated all studies with full or spot manual-scoring. For all software, the maze is divided into an outer rim (~56%) and center region (44%). Each target hole is segmented such that the hole is within the center of each zone. The “visit” zone and “entry” zone are defined to allow the software (or manual-scorer) to know when the mouse has found or exited the maze. In our maze, visits were counted if the mouse’s head approached the hole from the outside (open) edge, within 5 mm.

### Outcome Measures: overview

There are separate outcome measures for learning trials (to evaluate learning rate) and probe trials to evaluate retention and motivation. Primary outcome measures for learning trials (latency, distance, unique or total visits) should be predetermined to allow accurate hypothesis testing. However, with new disease models, screening for a variety of deficits is sometimes desired or the motivation behind a previously found deficit is not yet known. In this case, it may be warranted to evaluate each outcome as a hypothesis-generating screen. Regardless, the cost of a type 1 vs. type 2 error should be considered and noted when analyzing multiple behavioral outcomes.

### **Learning Rate** (Learning Trials):

#### LATENCY:

This is the time (seconds) it takes the mouse to find/enter the target hole. Latency is sensitive to confusion, orienting, freezing, grooming, napping, etc. but also impacted by motor deficits and overall speed.

#### DISTANCE:

The distance (meters) it takes the mouse to find/enter the target hole. It is less impacted by motor deficits but penalizes mice that prefer to follow the edge (thigmotaxis), even if they remember the spatial location. It is insensitive to changes in behavior due to increased freezing, orienting, or napping, when the mouse isn’t moving.

#### UNIQUE VISITS:

The number of holes checked once (range 1–10) needed to reach the target hole. Statistical chance is 5.5 holes visited. When averaged across multiple trials (one animal) or across multiple animals (one time-point), this outcome can test for statically “beating chance,” thereby demonstrating the group/mouse is using a cue other than random search.

#### TOTAL VISITS:

The total numbers of visits to holes (ranging 1–unlimited) needed to reach the target hole. Statistical chance (assuming zero working memory and no serial checking) would average 10. While “zero working memory” is highly unlikely, it provides a meaningful number against which to compare. For example, a mouse that consistently returned >10 times to the same arm would be demonstrating abnormal ***perseveration***, not impaired memory, as this many incorrect visits would be inconsistent with chance alone.

#### TRIAL-BY-TRIAL outcomes:

Latency, distance, unique visits and total visits are each different measures of how efficiently the mice finds the target. Typically, all measures decrease trial-by-trial and day-by-day, due to 1) **procedural learning** (understanding how the maze works and how to search more efficiently and move faster) and 2) **spatial learning** (remembering the actual location [or another cue] and using that information to approach the hole more efficiently). Trial-by-trial outcomes are highly variable due to innate curiosity, a feature and flaw of the Barnes (see discussion). A repeated measures ANOVA (or repeated mixed effects model if missing data) is appropriate assuming trials are not ended prematurely. If trials are ended prematurely, we recommend statistical consultation for non-normal data.

#### DAY-BY-DAY outcomes:

Latency, distance, unique visits and total visits can be averaged across trials within the same day (excluding the habituation trial) to reduce random noise and for illustrative purposes. Although power is slightly increased, the highest rate of learning is usually within the first several trials and differences between groups may be obscured by averaging data.

### Memory and Motivation Outcomes (Probe Trials):

1. The Barnes FIELD protocol includes 4 separate memory probes to detect evidence of spatial memory independent of procedural learning. Three methods for calculating probe trials were compared:
  A) **Percent Time in sector:** The percentage of time spent in the target sector calculated from the total amount of time on the maze. For a 10-hole maze, this is 3/10ths of the maze (target hole and two adjacent). For a 12-hole maze this is quadrants of the maze (target hole and two adjacent holes). This outcome is most commonly used for probe trials. It assumes mice will search more in both the precise and general spatial location.
  2) **Percent time at target:** The percentage of time spent investigating the more precise sector (containing only the target 1/10^th^ of the maze). Using a more precise sector is necessary to evaluate the effect of non-spatial cues (odor, intramaze cues) as it cannot be assumed the mouse would generalize to the nearby holes. Our recommendation is to evaluate the more general region (target plus both adjacent holes) to reduce variability, unless testing a non-spatial cue.
  3) **Total distance travelled** (hyper-activity, speed, perseverance): Total distance during the 90 second probe encapsulates the speed and motivation of the mouse. Normal behavior is to check the expected hole, and once found to be blocked, explore the rest of the maze to look for alternatives. A perseverative mouse might stay only at the expected hole and not move on, whereas a non-motivated or fearful mouse might fail to explore at all. Hyperactivity relative to control animals would include travelling much greater distance with or without successful exploration of the majority of the maze.

### STARR-FIELD OUTCOMES:

#### First barrier trial:

1. Exploration in response to novel barriers (same measures as subsequent trials).

#### Learning Trials:

1. Same measure as Barnes-FIELD (latency, distance, unique visits, total visits).
2. Cognitive flexibility: Rate of learning new location following each switch.

#### Final Probe trial:

1. **Working memory** errors (cumulative number of “errors” (repeat entries) made prior to exploring the entire maze).
2. **% time in each sector:** Relative balance of time spent at most recent (reverse target), relatively recent (new target) and originally learned location (Barnes target day 1–3). We recommend using the target hole plus one hole on each side (e.g., 3/10ths for a 10-hole maze).

### The Barnes-FIELD and STARR protocols in mouse models cognition:

We sought to test the usefulness of our protocol as a screening tool for evaluating learning, memory, executive function and behavioral flexibility across existing and new mouse models of cognitive change. Studies were performed by laboratories at the University of Iowa who requested assistance with cognitive evaluation of mice between 2020–2024. Each laboratory was taught the current protocol and used the same setup, making minor changes if needed for their experimental design. Importantly, teaching the task required only 1–2 hours and then the investigating laboratory performed it independently in most cases. Data presented here are reanalyzed from prior publications or with permission from manuscripts in preparation. Only details relevant to the analysis of the Barnes protocol are presented here.

#### 5xFAD:

8–9 month-old male and female heterozygous 5xFAD vs. littermate controls (Tg(APPSwFlLon,PSEN1 *M146L*L286V)6799Vas, Jackson Laboratories) were used to test the efficacy of the drug terazosin. Only water treated mice are presented here.

#### Kaolin injection as a model of Normal Pressure Hydrocephalus (NPH):

8-month-old C57BL/6J mice injected intraventricularly with saline and mice injected with kaolin to induce hydrocephalus as a model of normal-pressure hydrocephalus (NPH) [33]. Reanalysis was performed to allow comparable outcome measures as other experiments.

#### Aged Postpartum:

~12-month-old C57BL/6J females with and without a history of pregnancy were administered sertraline or water. Groups were collapsed across treatment in this analysis to focus on the outcome measures with postpartum condition as the manipulation. Full comparisons of all manipulations have been submitted separately.

#### Traumatic brain injury (TBI):

Sham mice vs. those with frontal impact were evaluated for apathy vs. hyperactivity on the probe trials to demonstrate a different outcome compared with our other models. Analysis of other aspects of this manipulation are in preparation.

## RESULTS:

There are two key obstacles preventing evaluation of training trials to assess learning rate in a spatial acquisition task; first, mice might use olfactory or visual cues to locate the target escape box during learning. Second, the literature is mixed as to which outcome measures (e.g., latency, distance, etc.) are most sensitive and specific to deficits in cognitive functions during these learning trials.

To address these obstacles, we tested whether mice with a reused escape shuttle would utilize subtle olfactory cues in the absence of spatial information to identify the target hole. Outcome measures were also compared across protocols to assess the sensitivity to detect differences in spatial learning, using one protocol (random location, no cues) to artificially model a group with a complete deficit in spatial learning.

We randomly assigned 3-month-old male and female wildtype C57B6J mice into three separate conditions, each with a different protocol. In the first protocol, designated “NEW-shuttle,” we changed any entered shuttle out for a new, clean shuttle and changed the target location for each trial to a random spatial location. By design, any improvements across trials would be procedural: defined as changes in search efficiency, velocity, etc.) as no other cues were present. In the second protocol, “SAME-shuttle,” for each mouse we used the same target shuttle for all trials across all 4 days. The shuttle was not cleaned for olfactory cues between trials, but each mouse had its own personal shuttle copy. The target spatial location was changed randomly for each trial so that by design, improvements across trials would represent normal procedural learning plus any benefit from the olfactory cue. For the third group, we used our proposed Barnes protocol, “FIELD-protocol,” which is directly adapted from typical Barnes spatial training, but with the benefit of printable individual shuttles. Washed escape shuttles are never used as the target more than once per >10 trials and moon caps hide the entrance so that only external spatial cues are available for learning.

### I: Improvements in distance and latency are primarily procedural, but the benefit of spatial cues is statistically detectable.

#### Latency:

**Figure 3:**
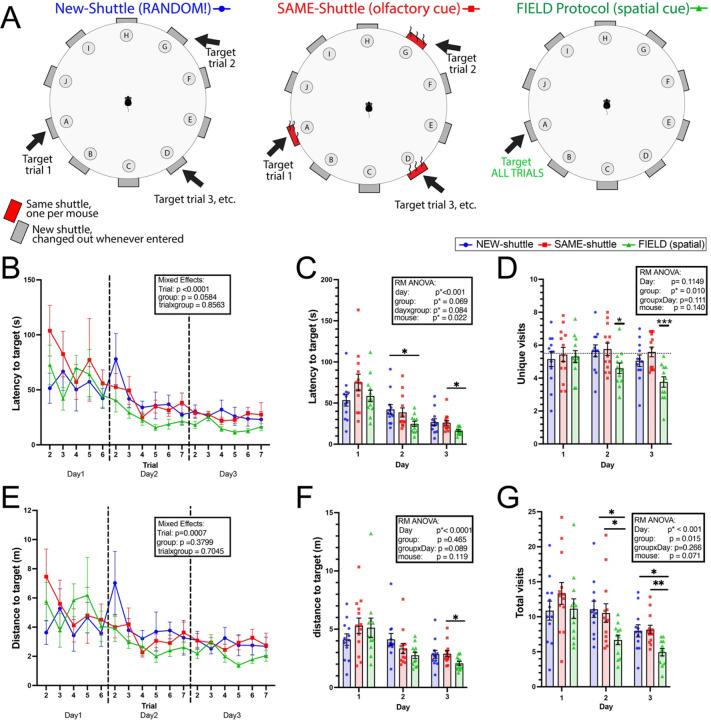
Procedural vs. Spatial learning benefits (A) Schematic of compared protocols. (B) Latency by trial across three days of training. (C) Latency averaged for each day. (D) Unique visits averaged for each day (E) Distance by trial across three days of training. (F) Distance by day. (G) Total number of visits (includes working memory error repeat visits). N = 12 (NEW), 13 (SAME), 12 (FIELD). Repeated measures (RM) ANOVA (C, D, F), Mixed-effect model (B, E) One-sample t-test against theoretical chance (5.5 visits, dotted line) (D). For planned comparisons (C, F, G), p-values are corrected for multiple tests (Tukey). *p<0.05, **p<0.01 ***p<0.001

First, we examined latency, the most commonly used outcome measure for learning trials [34]. Changes in latency across time provide an estimate of learning that encompasses multiple variables; it is influenced by time spent orienting and planning, as well as changes in speed, efficiency, search strategy, and spatial knowledge of the target location on the prior trial. All three groups showed significant changes in latency to find the target, including across individual trials (Fig 3B) and when compared across days (Fig 3C). A significant main effect of day was seen across groups, suggesting the mice gained significant procedural knowledge of how to improve on the task (how to search, moving more quickly, less lingering, less double checks). When comparing latency across all three groups, main effect of group did not reach significance (*p=0.0584 across trials, *p=0.069 across days), suggesting it is somewhat difficult to detect spatial learning from latency alone. Pre-planned comparisons of latency averaged over each day (Fig 3C) did suggest the FIELD group (with spatial cues) have a latency advantage of spatial learning that is statically detectable, but on inspection is notably less dramatic. By contrast, there was no difference between the OLD and NEW shuttle group, suggesting that if the mice use the olfactory cue to guide their search, it does not improve latency.

#### Distance :

Next, we examined distance as an outcome measure. Distance to target removes influences attributable to motor speed, which could be a confound for older mice or neurodegenerative models with motor features. However, it may also miss time the mouse spends orienting, planning, grooming or napping, which could indicate abnormalities. Similar to latency, distance to target across all groups showed a significant main effect by trial (Fig. 3E) and by day (Fig. F), improving over time.

### II: Unique visits provides statistical evidence for use of available cues.

#### Unique Visits:

While distance and latency are most classically used to evaluate learning trials, evaluating unique visits provides the opportunity to test if the group is performing better than statistical chance over the course of learning. We therefore hypothesized that our random “NEW-shuttle” group (with no other available cues) should (*when averaged over enough random trials*) visit 5.5 holes to find a target hidden amongst 10 identical holes.

As hypothesized, the NEW-shuttle group did not differ from the theoretical mean of 5.5 on any of the three days (one-sample t-test against hypothetical mean of 5.5), evidence that our 3-D printed shuttles and moon caps successfully hid extraneous cues (odor or visual) during learning trials. Interestingly, the SAME-shuttle protocol also did not differ from chance across days, suggesting mice did not generally use the odor cue to gain advantage in reaching the target. By contrast, by Day 2 and 3, the FIELD-protocol group differ significantly from chance (Fig. 3D: Day 2: *p < 0.05, Day 3: **p < 0.01). This provides statistical evidence that by the second day of training, the mice are using spatial cues to navigate. Furthermore, comparisons between groups using ANOVA suggests significant differences between the protocols. Thus, spatial learning may be more easily detectable using unique trials as an outcome measure, while latency and distance provide estimates that include both procedural and spatial learning.

#### Total visits:

Finally, we examined the number of total visits, which includes both unique visits (as above), as well as repeat visits, which could be indicative of perseveration or working memory errors (Figure 1G). Here, we found, much like distance, mice in all groups tend to complete the task with fewer visits to incorrect holes with each consecutive day (main effect day *p<0.0001). There was also a significant main effect of group (*p=0.0151), with pre-planned comparisons indicating larger improvements in the FIELD-protocol group (indicating benefit from use of the spatial cues) on Days 2 and 3 compared with the NEW-shuttle and SAME-shuttle groups.

### III: Olfactory cues are a potential confound; 3-D printed shuttles mitigate this risk.

Next, we used the probe trials to analyze whether the SAME-shuttle group had learned the odor cue, despite the lack of impact on learning trial outcomes. In probe trials across protocols, all shuttles were left in place, but entry was blocked by plastic grates on each shuttle to prevent entry. Importantly, no extra odor cues were added, there were still 10 identical shuttles present, and within SAME-shuttle groups, the shuttle is in a new random spatial location. Thus, the only cue available for the SAME protocol is that the target is a shuttle previously used by that mouse throughout training. Mice in the SAME-shuttle group whose shuttle box was placed in the location of the prior trial (because of random assignment) were excluded from this comparison.

**Figure 4:**
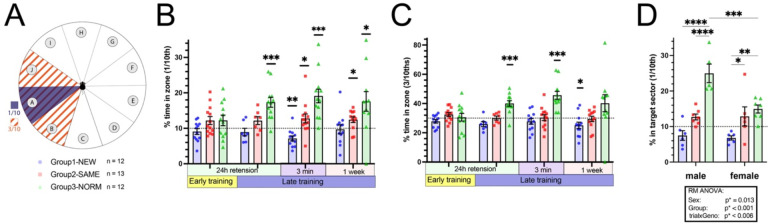
Intermittent probe trials for spatial acquisition and retention. (A) Schematic of 1/10^th^ versus 3/10ths of the maze to evaluate spatial memory. The odor cue will only be present in the 1/10^th^ sector (B) The percentage of time spent in 1/10^th^ of the maze following 24-hour retention at beginning of day 2 (after 5 learning trials), and day 3 (after 11 learning trials) and short-term memory at end of day 3 (after 17 learning trials), and 1-week retention. (C) The percentage of time spent in 3/10ths. (D) Comparing percent time (1/10^th^) between males and females on the 3-min retention trial (no sex differences were seen at other probes). N = 12 (NEW), 12 (SAME), 12 (FIELD). One-sample t-tests against theoretical mean of 30% (B) or 10% (C). (D) RM (Repeated Measures) ANOVA, with p values corrected for multiple comparisons (Tukey) *p<0.05, **p<0.01, ***p<0.001, ****p<0.0001.

Intriguingly, we found that mice can identify the olfactory cue of the randomly placed escape shuttle, and that they learn and retain over a similar timeframe as spatial cues. At the short-term memory probe and the 1-week long-term probe, mice in the SAME-shuttle group spent more than 10% of their time exploring the 1/10^th^ sector containing the target shuttle (Fig. 4B). By contrast, mice in the NEW group did not spend a greater amount of time in that target sector.

Evidence that the olfactory cue detected on probe trials was seen when evaluating 1/10^th^ of the maze (sector with one hole), but not detectable when evaluating 3/10^th^s of the maze (similar to commonly used “quadrant”). This may be due to the nature of the odor cue, which would only exist at one target hole. With training using a spatial cue in the FIELD-protocol, there was evidence of increased time spent regardless of whether a more precise (1/10^th^) or broad (3/10^th^) sector was used (4B and 4C). Use of a narrow vs. more spatially generalized sector of the maze for the probe trial may be sensitive to different aspects of cognition and was explored further in the case studies below.

### IV: Interleaving probe trials allows evaluation of early and late acquisition as well as long-term (24h and 1 week) and short-term (3 min) retention.

Interleaving probe trials allows testing of memory over the course of learning (during acquisition) and retention over short and long intervals. Analysis during acquisition is important as many models are able to learn and retain the task, but learn procedurally and/or spatially at a slower rate. Protocols sometimes omit probe trials over concern for extinction of learned spatial cues. Although we did not directly test learning with and without the probe trials head-to-head, we were usually able to detect evidence of spatial memory in control groups across probes (Fig. 4B–C, Fig. 6) despite including up to four separate probe trials. In our shuttle odor experiment, the spatial learning FIELD group showed evidence of memory retention at all probes except the first day, demonstrating the time-course of this acquisition.

### V: Outcome measures are sensitive to differing aspects of learning, but generally agree on overall findings.

**Figure 5:**
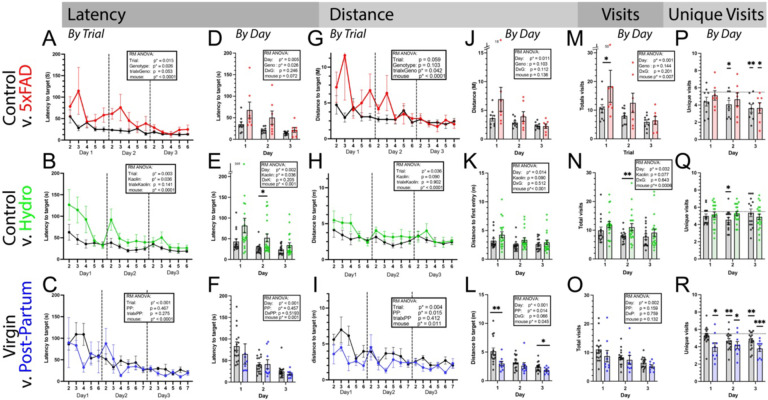
Outcome measures on learning trials across three models of cognitive impairment. 5xFAD (9–10months old, red), hydrocephalus (8-months-old, green) and post-partum females (1 year, blue) compared with age-matched and/or control injection controls (black). **Latency** by trial (A-C) and by day (D-F) shows main effect of trial/day across conditions and experiments, demonstrating improvements to find target. **Distance** to target by trial (G-I) and by day (J-L) shows very similar time-course but may be sensitive to slightly different factors between groups. **Total visits** by day (M-O) and **Unique visits** (P-R) allows comparison to chance. Mice with zero working memory should by chance average 10 total visits. Above that indicates purposeful repeat visits, below represents good working memory. Mice with no spatial memory should average 5.5 unique visits, with average below providing evidence for spatial acquisition (P-R). Repeated Measures (RM) ANOVA used for all comparisons except in the case of missing trials. Pre-planned comparisons by group were only done on day-by-day analysis and were corrected for multiple comparisons (Tukey). For unique visits, each probe was separately compared versus chance (5.5) using one-sample t-test. p*<0.05. **p<0.01, ***p<0.001.

We evaluated latency (by trial and by day, Fig 5A–F), distance (by trial and by day, Fig 5G–L), and total visits by day (Fig 5M–O) in three models of cognitive changes performed by three separately laboratories for evidence of learning rate and differences between groups. Across all experiments and outcome measures there was a significant effect of day or trial (respectively) or interaction by trial. This indicates changes in outcome as a function of time, demonstrating learning. Importantly, the change likely represents both procedural and spatial learning. When comparing between experimental manipulations, 5xFAD mice showed evidence of differences in latency (across trial or day), with a significant interaction when evaluated with distance by trial (Fig 5G). Within the NPH manipulation, latency again was slightly more sensitive to differences, but in general, all outcomes showed the same trends. In post-partum mice, distance was more sensitive to differences, both across trials and days (Fig 5I, L), and could suggest a different aspect of behavior is altered, although in general, the trends remain the same across outcome measures.

**Figure 6:**
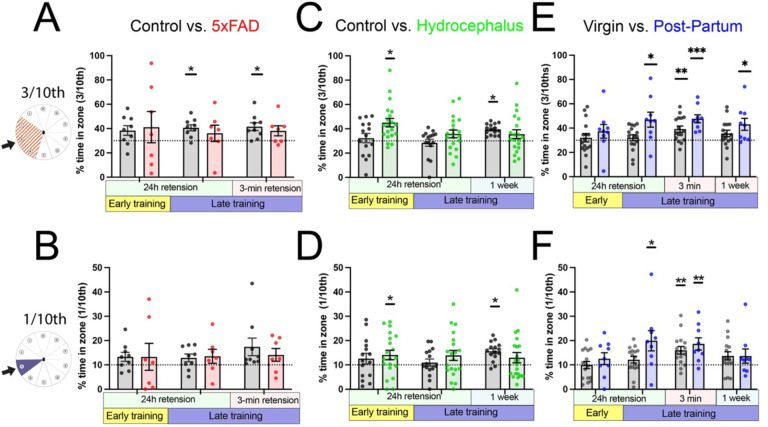
Probe trials provide a statistical test of spatial acquisition. (A) 5xFAD mice as a group fail to show evidence of spatial learning compared to chance, which age-matched controls show evidence at a late 24h retention and a 3-min retention probe. No 1-week probe was done. (Control: n = 9, 5xFAD: n = 7). (B) Neither group shows statistical evidence when a more restricted sector is analyzed. (C) Controls in the hydrocephalus experiment only show evidence of spatial acquisition at 1-week (no 3-min probe was done), whereas the hydrocephalus manipulation shows evidence on day 1. (D) Conclusions remain the same regardless of precise vs. broad sector of comparison. (Control: n = 15, Hydrocephalus: n = 20). (E) Postpartum mice show evidence of spatial acquisition in all probes following the first day. (E) There are slight differences in interpretation depending on sector evaluated (F) (virgin n = 16, post-partum: n = 9). All evaluated with one-sample t-test against theoretical mean of 30% (A, C, E) or 10% (B, D, F). p*<0.05. **p<0.01, ***p<0.001.

Given our findings in our shuttle experiment, we also evaluated the number of unique visits. Evaluation of unique visits acts as a complement to the probe trials (discussed below) but allowing comparison against statistical chance. Notably, this measure agrees relatively reliably with the probe trials (Fig 6) even though it is derived from independent trials (probe trials are not included in learning analysis in order to avoid double analysis). We found evidence that 5xFAD mice (as a group) show evidence of spatial learning on average by day 3 (Fig 5P, whereas their littermates as a group show evidence by day 2). Importantly, as by-day analysis allows evaluation of individual mice, the consistent variability in the 5xFAD mice is notable, with some mice showing clear acquisition early on. In the NPH experiment, the saline-injected group showed evidence of performance better than chance at day 2 but failed to show at day 3. This matches the probe trials for that day (Fig 6C–D), but the reason is unclear. Notably, spatial acquisition was evident at the 1-week probe.

Finally, 1 year old post-partum mice showed evidence of spatial acquisition on all three days, demonstrating early learning compared with virgin mice of the same age. Importantly, virgin mice did show evidence by day 2, similar to our other control groups (Fig 5R).

### VI: Probe trials evaluate spatial acquisition and retention across learning.

We next evaluated probe trials across learning to test for spatial acquisition. This includes two 24 hour retentions probes at the beginning of day 2 (after 5 learning trials) and day 3 (after 11 learning trials). The 3-min retention and 1 week retention are both performed after the final learning trial (17 learning trials). Not all experiments had all possible probe trials (Fig 6). We evaluated all groups with a more precise (1/10^th^) vs. broader (3/10^th^) spatial location in order to determine if this choice might impact results in the literature. In general, more groups showed improvement compared with statistical chance when using 3/10^th^, suggesting it may be more sensitive to detecting evidence of spatial learning. 3/10ths (one hole on each side of the target) should be similar to “quadrants” typically used in the literature when hole numbers are divisible by 4. 1/10^th^ may be more appropriate in certain situations (as in odor cue in Fig 4B, but importantly, the general trends and remain similar regardless of outcome chosen.

### VII: Probe trials are subject to multiple motivational factors:

**Figure 7:**
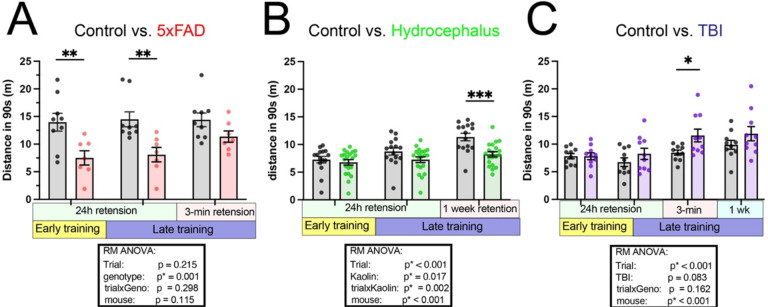
Total distance travelled in 90-seconds on probe trials is sensitive to behavioral differences. (A) 5xFAD mice show significantly reduced distance travelled across probe trials (main effect genotype) (n=9,7). (B) Hydrocephalus model shows a group and group by trial interaction, suggesting the Kaolin injected mice does not increase distance travelled as learning occurs (n=15,20). (C) A mouse model of TBI shows increased distance travelled during the 3-min retention (n=10). p*<0.05. **p<0.01, ***p<0.001. Tukey’s correction.

Although the outcome of the probe trials (percent time in sector) is theoretically less influenced by motor speed compared with latency on learning trials, it is not immune to motivation and motor speed differences. A mouse the moves more quickly or is more efficient in exploration, will take less time to explore a sector, and therefore might spend relatively less time in the target sector over 90 seconds, despite good spatial acquisition. Evaluating other behavioral outcomes can help detect possible motivational or motor difference between groups. For this reason, we evaluated total distance travelled on the probe trial and found it was highly sensitive to differences between groups. 5xFAD mice showed significantly reduced total distance in 90 seconds across probe trials (Fig 7A), with comparisons suggesting this effect was less notable in the 3-min probe, which may indicate that during longer retention intervals 5xFAD mice forget the existence of a hole or are more likely to “give-up.” In most mice there is an increase in distance travelled as training precedes, but this change across time is different in the hydrocephalus mice compared with controls (Fig 7B, trial x Kaolin, p* = 0.002). The differences (or lack thereof) across trials suggests that motivation rather than motor deficits may play a role. We also include an example outcome with increased activity during the probe (Fig 7C): a mouse model of TBI shows increased distance travelled at the 3-min probe on pre-planned comparisons, demonstrating differences can occur in both directions. Additionally, by inspection, note the major differences between control groups across experiments, which may relate to genotype, strain, age, experimenter or the additional behavioral tests completed by each group.

### VIII: Spatial Training and Rapid Reversal (STARR) can be performed after a standard Barnes maze to challenge more rapid, executive functions.

**Fig 8:**
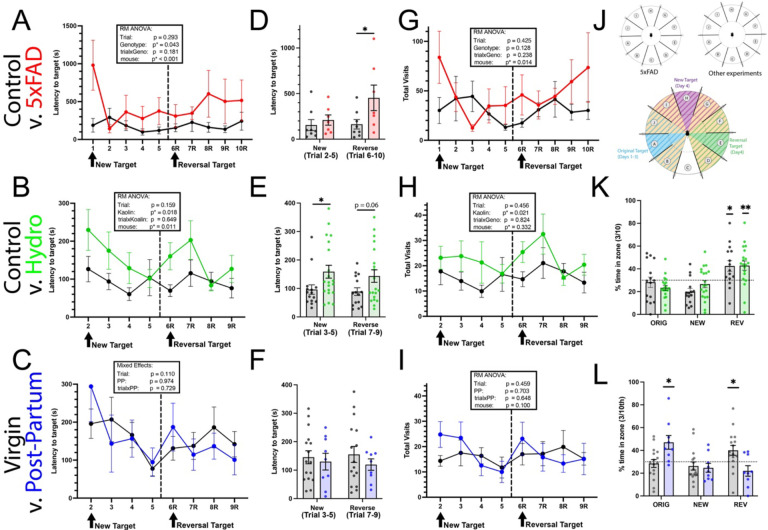
Spatial Training and Rapid Reversal (STARR) challenges rapid learning, working memory and flexibility. Latency to find a new target was sensitive to behavioral differences due to 5xFAD (A) and hydrocephalus (B), showing a main effect of group. 1-year-old post-partum vs. virgin female mice did not differ on their ability to learn the STARR (C), but both groups demonstrate the latency “bump” usually seen when the target is moved at trial 6R. Averaged latency (excluding trials with a new target) allows better inspection of differences between individual mice (D-F). Total visits (G-I) includes both primary and working memory errors. Intriguingly, a 90-second probe trial provides evidence that control and hydrocephalus mice remember the most recent target (K), whereas a 5-min probe trial (L) suggests post-partum mice may be able to remember the previously acquired (day1–3) location, whereas virgin mice remain more interested in the most recent target. p*<0.05. **p<0.01

The STARR maze incorporates barriers entice mice to return to the center, increasing the likelihood of working memory errors and discouraging serial checking. We evaluated outcomes over three experiments with relatively similar versions of the protocol (Fig 8). In both 5xFAD and hydrocephalus groups, the presence of the barriers and rapidly changed spatial targets unmasked deficits that had been less evident by the end of the third day of Barnes training (Fig 8A and 8B). Post-partum and virgin mice, on the other hand showed similar latency during the STARR (Figure 8C). A recent addition, the probe trial, provides valuable additional information regarding acquisition during this task. Hydrocephalus mice were run on a 90-second probe with a 3-min delay, and both groups showed evidence of remembering the most recent target. Due to differences in protocol, the post-partum mice were run on a 5-min probe trial. Intriguingly, the post-partum mice showed preference (and therefore memory) for the originally acquired location, whereas the virgin mice appeared to prefer the most recent (Fig 8L). This may be due to differences in acquisition (post-partum mice had better retention in the 1-week probe), or behavioral flexibility.

## DISCUSSION

We modified a classic, widely used spatial acquisition and memory task to distinguish the types of learning and motivation features and improve reproducibility by using 3D printable shuttles and a home-cage attachment that can be made or ordered by any laboratory. Importantly, these findings allow improved interpretation of the Barnes even for protocols that vary from the one recommended here.

To test the utility and necessity of our 3D printed shuttles, we tested the impact of a reused escape shuttle, as is the standard for many commercial systems (e.g., TSE Systems, Maze Engineers). We found the reused shuttle did not change latency or distance outcome during learning trials. This is either because the mice do not use the odor cue to improve their search, or, if it is used, it does not improve any of the outcome measures (latency, distance, unique visits, or total visits) when compared with random chance. By contrast, our probe trials provide clear evidence that mice could detect the odor of the reused escape shuttle, remembering it even a week later. Thus, in theory, the presence of the odor cue during spatial learning could help or hinder retention of the spatial location in ways that might differ by experimental model. Since in our experiment the odor cue was tested under the condition of a random spatial location, and was not tested with neurodegenerative models, it is important to highlight that our data only provides evidence that it is *possible* for mice to acquire this subtle cue, and thus prudence is indicated. Importantly, our 3D printed shuttles and moon-caps (blocking visual cues) mitigate this risk while retaining the low-stress, naturalistic aspects of the Barnes that can make it preferable to tasks like the Morris Water Maze.

Next, we took advantage of the setup of the experiment (spatial location vs. random location) to evaluate commonly used outcome measures to determine which aspects of learning can be identified from learning and probe trials. Each outcome measure has distinct sensitives and potential confounds. For the purposes of proper statistical testing, a single outcome measure for each portion of the protocol (learning trials and probe trials) should be chosen ahead of time. Additional exploratory outcome measures can then help aid in interpretation of results.

Our study demonstrated that latency and distance improvements due to spatial learning are minor compared with gains across trials due to non-spatial learning, which could include procedural learning, memory of the task ‘in general’, motor learning, search strategies, working memory improvements and motivational improvements. This has been previously reported in the literature [35; 36], but is often ignored when latency is listed as the primary outcome measure. The benefit of spatial learning may become more detectable as training duration increases. However, in many of the experimental groups studied, the differences between groups dissipate as training time increases.

Thus, it is essential to understand the contribution of both procedural and spatial learning during early training.

The finding that latency and distance outcomes are highly sensitive to non-spatial learning does not make them less useful. Use of only the probe trial (to gauge spatial) may miss substantial differences in this naturalistic learning process. Importantly, latency and distance are sensitive to multiple types of non-spatial learning that would be difficult to evaluate on probe trials, including procedural, episodic and working memory deficits. Mice must remember 1) that the escape hole even exists, 2) how to find it, 3) how to be efficient, including improvements in working memory errors (checking the same hole/location twice). On the other hand, unique visits as an outcome measure during learning trials allow identification of the benefit of spatial learning and direct comparison with chance. With these two outcomes, we can screen neurodegenerative models with better insight regarding potential underlying causes of differences found.

Evaluation of several different models of dementia and aging using our modified task illustrate the utility of this careful consideration for outcome measures and use of both probe and learning trials. 5xFAD mice are a model of beta-amyloid build up and have previously been shown to have deficits in hippocampus-dependent spatial working memory using Y maze task [37]. Studies using different versions and protocols of Barnes maze have been used as evidence that 5xFAD mice also have deficits in spatial learning and memory. Studies have reported lower success rate to find target, higher escape latency, working memory errors and increased use of non-spatial search strategies [38; 39; 40]. Our study did find significant deficits in latency/distance, as well as deficits in unique and total visits, suggesting the possibility for deficits in both spatial acquisition and memory, as well as other aspects of learning (procedural/working memory). Most strikingly, 5xFAD mice improved to near wild-type performance with extended training, a finding that is supported by other published studies. For example, depending on the outcome measure, differences between 5xFAD and WT mice were no longer evident by the 2^nd^ or 3^rd^ day of training in one study [41]. Thus, evaluation of learning trials and intermittent probes is a compelling and useful tool to detect these clinically important early training differences. Of note, we were also able to “bring back” deficits in overtrained mice through the use of the STARR (Spatial Training and Rapid Reversal) protocol, which requires rapid acquisition of a new location as well as behavioral flexibility (see further discussion below).

Aspects of our protocol were evaluated in several other models of non-Alzheimer’s dementia and aging. In multiple cases we found latency to be the most sensitive to differences between the groups. However, it also highlights that these differences could be attributable to multiple distinct deficits or motivational changes that may need further evaluation.

Mice with intraventricular kaolin injection as a model of normal pressure hydrocephalus were shown to have increased latency to find the target during learning trials [33]. This finding could be due to reduced motor speed of these animals. However, corroborating behavioral studies in their published work, evaluation of distance and total visits on the Barnes FIELD protocol demonstrated a deficit in learning the task: NPH mice have more total visits compared with saline injected mice. As this difference is present in total visits but not unique visits on the third day of learning, this could indicate a working memory deficit (or perseveration) rather than a deficit solely attributable to spatial memory. Thus, further studies investigating that possibility could be fruitful.

We also examined data from 1-year old female mice with and without a history of pregnancy. In this case the findings highlight a difference in learning rate, with the most notable differences seen in distance travelled during the first day of learning. This is corroborated by two separate indicators: 1) evaluation of unique visits during learning trials, which shows postpartum mice differ from statistical chance on the first day, while virgin mice perform at chance and 2) the separate 24-hour probe trial at the beginning of day 3 shows post-partum mice have acquired the spatial location, whereas virgin mice do not show evidence of acquisition by this timepoint. Importantly, both groups show evidence of spatial learning in the 3-min short-term memory trial at the end of the third day, as well as retention at 1 week, highlighting the importance and sensitivity of examining along the learning curve to detect differences.

While days 1–3 of our protocol improve interpretation of learning rate and memory retention, these measures may still be influenced by other motivations. We found that total distance travelled on the probe trial could be an important measure to help provide context to the results. Total distance travelled on the probe is theoretically influenced by multiple factors: hyperactivity/hypoactivity, apathy/perseverance, curiosity and exploration vs. fear, as well as declarative memory. Across our model animals, there were differences in distance travelled on the probe trials, and these differences tended to be consistent across probes suggesting they are true differences in the groups. It was notable that these differences were not evident in standard open field testing (when available). For example, hydrocephalus mice showed the same distance travelled in open field [33], but clear differences on our probe trials.

It is important to consider how this context impacts interpretation of spatial learning percentages on probe trials. Consider a hypothetical mouse that goes to the target, stops, and remains there for the entire duration. This mouse would be labeled as having “strong” spatial memory using percentage of time spent but is arguably abnormal in behavior; they would fail to explore alternative hiding places in the face of a blocked tunnel. Similarly, differences in activity can alter percentage time at sector and may or may not relate to spatial memory. For example, male and female mice performed similarly for latency and distance (not shown) but showed a statistical difference during the 3-min short term memory trial, although both perform better than chance. It is for this reason that we recommend evaluating probe trials with a “yes or no” statistical test against chance and visually inspecting the data. Comparing the magnitude above chance may not reflect better spatial memory.

Finally, we developed a test for behavioral flexibility and rapid acquisition that can be added after any traditional Barnes protocol. As noted above, group differences are often detectable in early training but become less apparent with extended training or overtraining. We designed and tested the one-day Spatial Training and Rapid Reversal: STARR-Field maze as an add-on to the Barnes maze. It allows assessment of spatial location acquisition over a rapid time frame, using four trials at a location distinct from the one originally acquired, followed by four trials at a third location. We have previously published evaluation of a Parkinson’s model of cortical synuclein using this task without barriers, showing slowed learning of the reversal target in this model [42]. In that study, we were able to repeat the protocol multiple times, including in the setting of injection with lipopolysaccharide (LPS). Here, we tested the use of barriers to prevent serial checking and allow for evaluation of working memory errors, similar to a radial arm maze.

This STARR-FIELD add-on protocol adds several important new features. The introduction of the barriers adds novelty to the environment and disrupts previously acquired search strategies. The first trial of day 4 within the barriers is included as part of the analysis (unlike the habituation trial on day 1). In general, mice recognize the task and do not require habituation or assistance. This is true for most mice, but as shown, the large increase in latency only on trial 1 for 5xFAD transgenics (Fig 8A) may suggest this group forgot the existence of the escape hole, or alternatively, were more reactive to novelty.

Importantly, the differences in latency that had nearly dissipated by the third day of training in 5xFAD and NPH groups become evident again just with the introduction of the barriers and new spatial location. An early iteration of our protocol had rapid reversal learning as day 4 (no barriers) and added barriers for working memory and new location learning as day 5. Although the protocol succeeded, very few labs were able to run this long a protocol. For this reason, we have attempted to optimize a combined 4^th^-day add-on protocol using both reversal and barriers. We expect this add-on protocol can be further optimized in future research as we expand to additional models of cognitive impairment. Refining rodent behavioral paradigms remains crucial for both basic and translational research. As drugs or interventions must prove efficacy in animals models before they can be considered for human use [43], it is imperative that outcome measures are well understood.

Overall, our novel STARR-FIELD protocol provides important new opportunities for the widely used Barnes maze. Findings in our 5xFAD and NPH cohorts on the STARR protocol support differences identified in the Barnes. Intriguingly, despite increased latency on the STARR, the probe trial at the end of the protocol suggests NPH mice were able to remember the most recent spatial location over the short interval. Thus, deficits in latency in this model may be more associated with some non-amnestic features, such as learning rate, search efficiency or working memory errors, in addition to deficits in long-term memory retention that were seen in the 1-week probe. Future work with a longer final probe trial will better help to distinguish between non-amnestic features and identify working memory errors.

### Limitations:

Limitations and remaining gaps are addressed throughout the manuscript. In particular, protocols were adapted and modified over the course of multiple years, with each cohort run under our supervision by separate investigators adapting to the needs of their experiments. Thus, caution must be taken comparing between cognitive models presented, especially since groups were not interleaved and control groups show striking differences. Similarly, we are not able to rigorously test all possible permutations for the protocol when creating the final recommendations, and limitations to how these choices were made are noted in the detailed protocol. Finally, human run mouse behavioral studies will never achieve the low variability of repeated, automated testing. However, operant chambers and in-cage testing are costly and will not replace manual studies for some time. Furthermore, although more variable, we believe there will always remain a benefit for naturalistic tasks observed by humans to allow direct discovery of unexpected behavioral findings and better interpretation of more rigid outcome measures from other tasks. As with direct interaction with human patients, there remains an important role for direct observation in mouse models.

Despite the mentioned limitations, we present evidence for useful modifications to the classic Barnes maze to improve and clarify interpretation of outcome measures for spatial learning, retention, non-spatial and procedural learning and motivation. Our findings have important implications for interpretation of both old and new versions of spatial acquisition tasks and provide steps to build this accessible cognitive screen at low cost. These features will improve rigor and reproducibility across neuroscience laboratories using behavioral outcomes.

## Supplementary Material

Supplement 1

Supplement 2
