# Supplementary figures and images for: Renovating the Barnes maze for mouse models of Dementia with STARR FIELD: A 4-day protocol that probes learning rate, retention and cognitive flexibility
Source: bioRxiv. 2024 Dec 1:2024.11.30.625516. Preprint. [Version 1] doi: 10.1101/2024.11.30.625516 (PMC11623659; doi:10.1101/2024.11.30.625516)

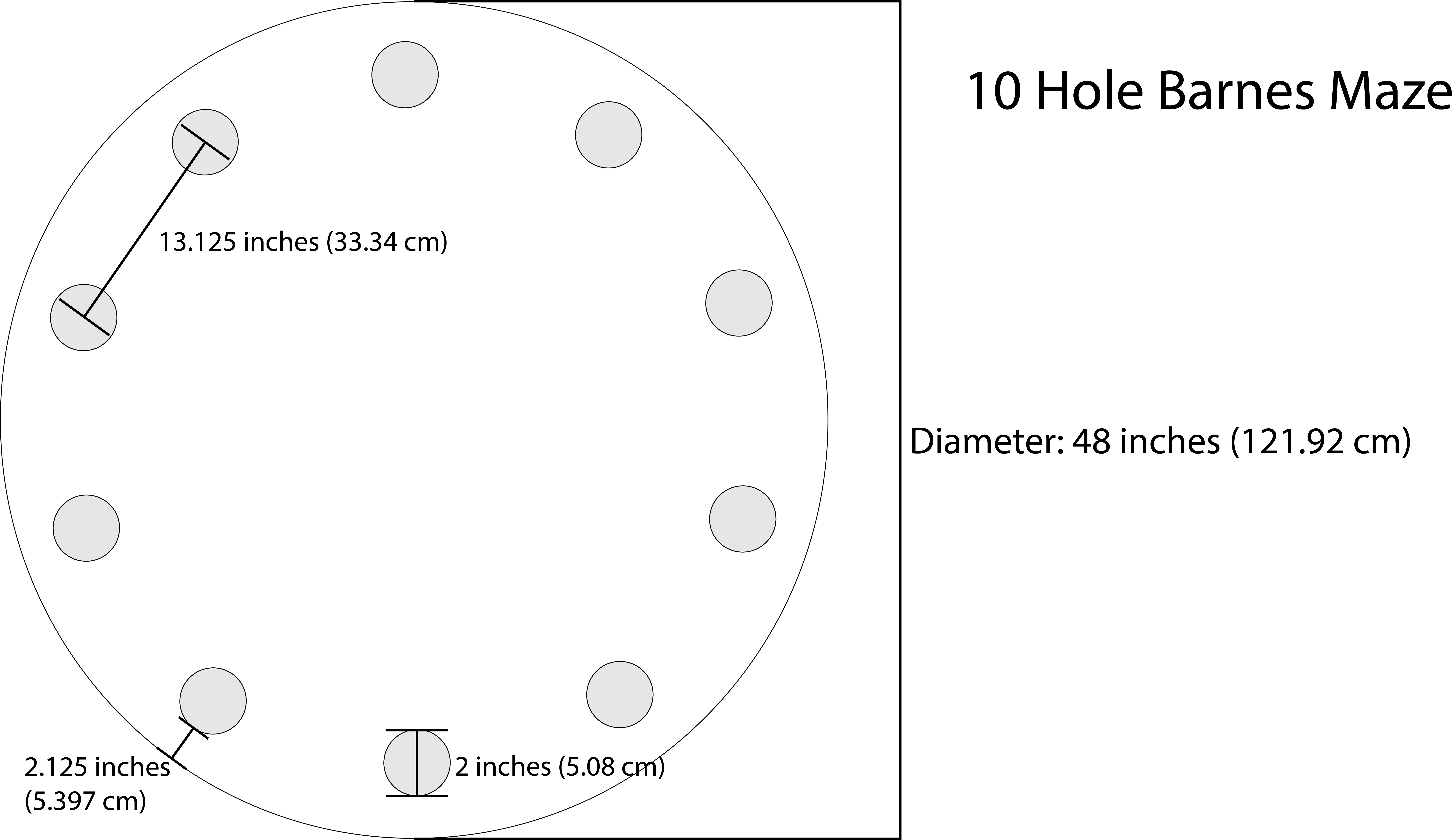

Supplement: Supplement 1 [file media-1.jpg]

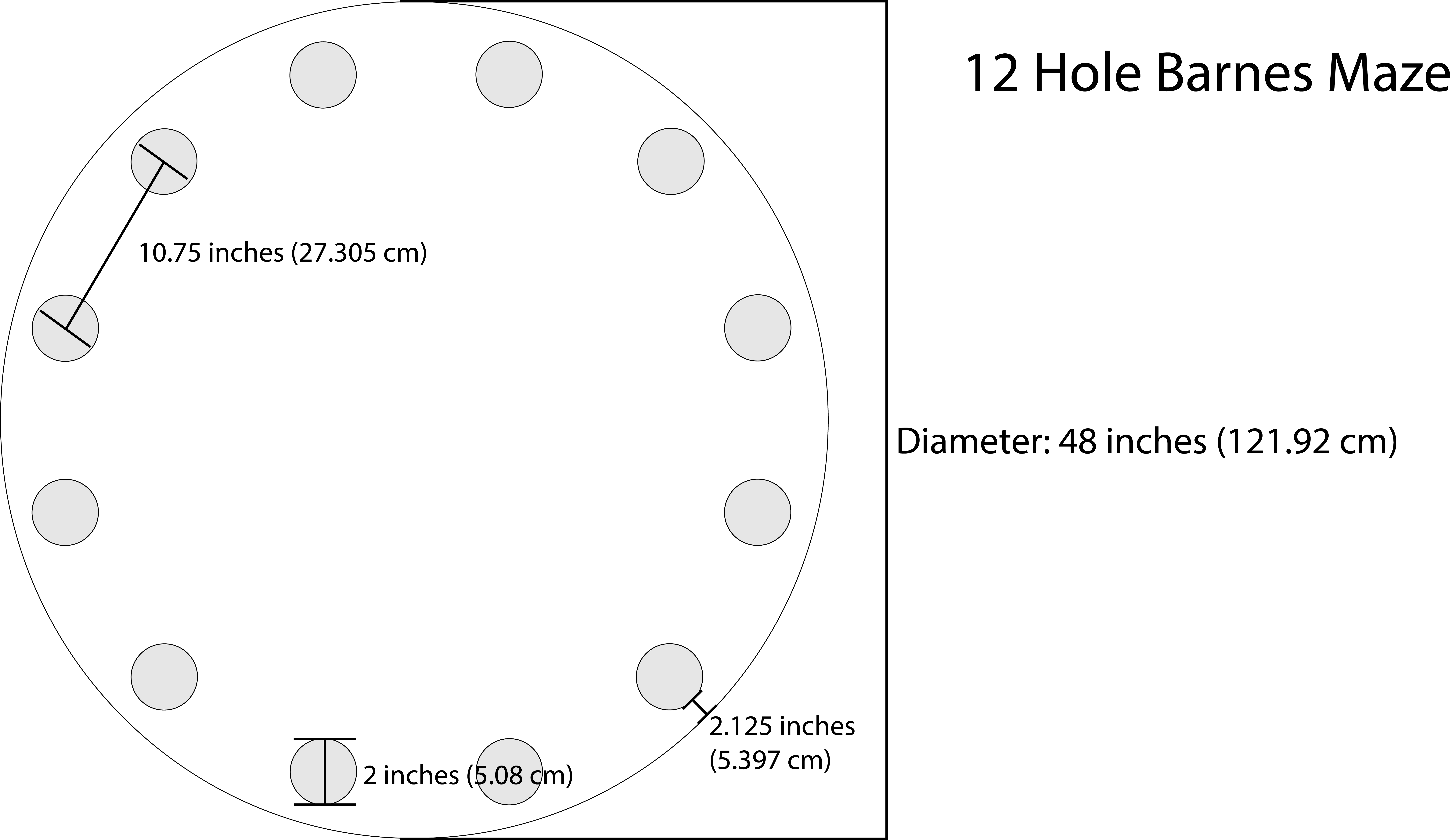

Supplement: Supplement 2 [file media-2.jpg]
